# Wildlife-vehicle collisions in Lanzarote Biosphere Reserve, Canary Islands

**DOI:** 10.1371/journal.pone.0192731

**Published:** 2018-03-21

**Authors:** Gustavo Tejera, Beneharo Rodríguez, Carlos Armas, Airam Rodríguez

**Affiliations:** 1 Canary Islands’ Ornithology and Natural History Group (GOHNIC), La Malecita s/n, Buenavista del Norte, Tenerife, Canary Islands, Spain; 2 Piedra Viva 26, San Bartolomé, Lanzarote, Canary Islands, Spain; 3 Department of Evolutionary Ecology, Estación Biológica de Doñana (CSIC), Avda. Américo Vespucio 26, Seville, Spain; Sichuan University, CHINA

## Abstract

Insular wildlife is more prone to extinction than their mainland relatives. Thus, a basic understanding of non-natural mortality sources is the first step in the development of conservation management plans. The Canary Islands are an important tourist destination due to their unique climate and rich scenery and biodiversity. During the last few decades, there has been significant development of urban areas and busy road networks. However, there have been no studies describing the effects of road mortality on wildlife in this archipelago. We describe the temporal and spatial patterns of wildlife roadkill in Lanzarote (UNESCO Biosphere Reserve), using counts from cars for an entire annual cycle. A total of 666 roadkills were recorded (monthly average of 0.09 birds/km and 0.14 mammals/km) comprising at least 37 species including native birds and introduced mammals. Seasonal abundance, richness and diversity of roadkills showed a high peak during summer months for both mammals and birds. GLMs indicated that accidents (including birds and mammals) have a higher probability of occurrence close to houses and on roads with high speed limits. When analysed separately, mammal kills occurred in sectors with high speed limits, close to houses and in areas surrounded by exotic bushes, while bird roadkills appeared in road sectors with high speed limits, close to houses and low traffic volume. Our findings highlight that roads are a potential threat to native birds in the eastern Canary Islands. Detailed studies on the local population dynamics of highly affected species, such as the Houbara Bustard, Eurasian Stone Curlew, Barn Owl or Southern Shrike, are urgently needed to determine whether these levels of road mortality are sustainable.

## Introduction

Roads have dramatically increased during the last several years, both in natural and urban areas, to satisfy transportation needs [1]. The ecological effects of roads include habitat loss or fragmentation, discharge of toxic gases and substances such as oils and waste, acoustic and light pollution that significantly affects the behaviour and biology of several species, facilitation of invasive exotic species, barrier effects for movement-limited species and, above all, direct mortality by collision with vehicles [2–4]. However, these linear infrastructures may also have positive effects on some bird species, by reducing predation, and providing foraging habitat, perches for hunting activities, or secure nesting sites [5].

Literature about factors explaining wildlife-vehicle collisions has increased in recent decades. Many studies highlight the importance of speed limit, traffic volume or characteristics of road margins on the occurrence of roadkills [6–10]. Some animal groups, such as large mammals or owls, are more prone to vehicle collisions due to their particular biology and behaviour. The number of victims is astonishingly large; for example, estimates range between 89 and 340 million birds killed annually by road casualties in the U.S. alone [11].

Usually species with low population sizes or local abundances, small range sizes and specialized habitat preferences have an unfavourable conservation status [12,13]. The life history traits of insular fauna are mediated by the so-called insular syndrome characterized by small and isolated populations, low reproductive rates and high adult survival [14]. As a consequence, island populations are more susceptible to human impacts than their mainland relatives [15,16]. Indeed, 37% of critically endangered species are confined to islands despite the fact that island surface area is only 5.3% of the Earth’s land area [17], and most island extinctions have been caused by anthropogenic impacts [18].

The Canary Islands constitute an important biodiversity hotspot within the Western Palearctic [19], and are host to a high number of endemic plants and animals [20,21]. For example, with regard to Class Aves, at least 5 full endemic species and around 35 subspecies occur in the archipelago today [22]. However, many Canarian endemic vertebrates have become extinct or critically endangered since the arrival of the first humans, around 2,500 years ago [23–25]. Several studies, focusing especially on seabirds and raptors, describe and quantify the effects of particular anthropogenic threats in this archipelago, such as collisions with manmade structures, poisoning, disorientation due to light pollution, direct persecution and habitat loss or modification [26–33]. However, as far as we know, no specific studies have dealt with road mortality on the Canary Islands.

Although nearly half of the area of the Canary Islands is legally protected for nature conservation, the existing policies promote high economic growth, mainly associated with the tourist industry, leading to the degradation of the landscape and other natural resources [34–36]. These islands support high resident human densities (282 people/km^2^) and are visited by around 11.7 million tourists each year [37]. In this scenario, fragile insular ecosystems are suffering unsustainable impacts from the massive development of urban areas and road networks [34,35,38–40]. It is logical to predict that many animal populations will become threatened or endangered in the near future. Thus, there is a need for studies quantifying the effects of particular sources of mortality. The main goal of this study is to analyse the temporal and spatial distribution of road casualties on the wildlife of Lanzarote, Canary Islands. To reach this aim, we assess species composition and identify the main road characteristics affecting road casualties. In addition, we estimate the number of roadkills and the impact on local populations of the most affected or threatened species.

## Material and methods

### The study area

**Fig 1:**
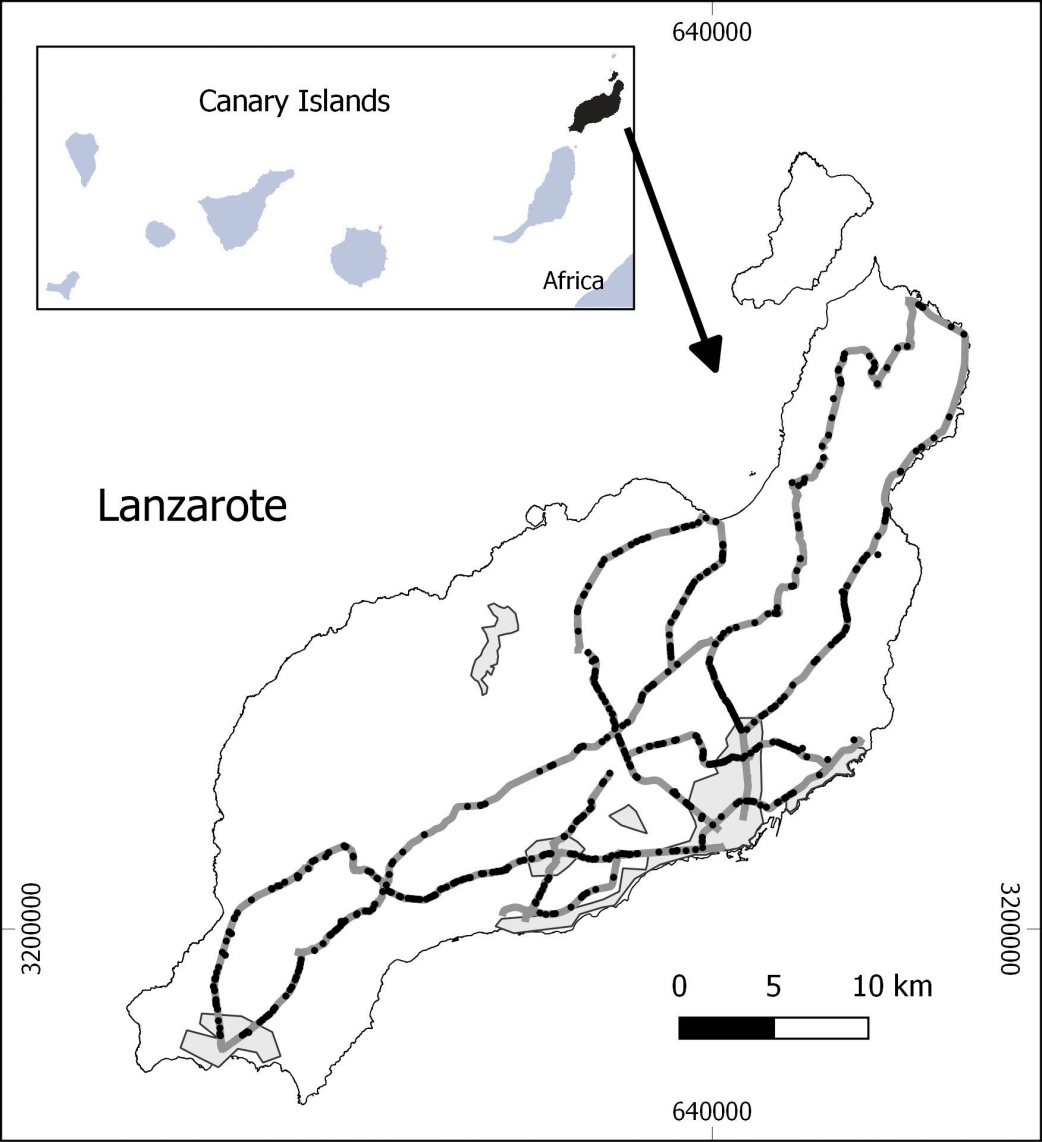
Location of Lanzarote Island and road network (in dark grey) studied during November 2010-October 2011. Grey areas indicate the most important cities and towns. Black points on the road network indicate roadkill locations.

The study was conducted on Lanzarote, the northernmost island of the Canary archipelago (Fig 1). Lanzarote Island has been a UNESCO Biosphere Reserve since 1993. Excluding the Famara massif (northern part of the island), the majority of the island surface (846 km^2^) is dominated by low and flat relief with scattered volcanic craters and related badlands. In general, rain is scarce and vegetation is dominated by low and sparse plants [41]. The local human population is around 143,209 people (in 2015), mainly concentrated on the southeast coast [37]. Today, the economy is highly dependent on tourism, attracted by a unique and fragile landscape, with agriculture and fishing activities in decline [42]. Each year around 2.1 million tourists visit the island, a figure about 15 times larger than Lanzarote’s resident population. Tourism has led to a rapid growth of urbanized areas and road networks. The private car fleet is composed of around 0.6 cars/inhabitants, and the road network (457 km in 2007; [43]) is highly saturated, particularly near tourist and urban areas [42,44].

Lanzarote is host to one introduced frog, three endemic species of reptiles, 47 species of breeding birds (44 native and three introduced) and seven mammal species (one endemic shrew and six introduced species) [45]. The island is also visited during the winter and passage season by migratory birds that usually come from Europe [46].

### Road surveys

The road surveys (*n* = 24) were conducted twice a month (in the first fortnight of each month in one direction and in the opposite in the second) for a whole annual cycle, starting the second fortnight of November 2010 and finishing the first fortnight of November 2011. In each survey, a total of 241.6 km of island roads were inspected (52.8% of the total road length on the island), such that the whole car census covered a total of 5,798.4 km. Surveys were conducted by a person driving a car at the minimum speed limit (20–50 km/h) and looking for dead animals on the road or in its vicinity. When traffic allowed the observers to stop the car, they approached the carcass to identify it to the species level. In some instances, photographs or biological samples (feathers, fur, or osseous remains) were taken to identify roadkills in the lab using field guides or reference collections. Taxa nomenclature follows Gutiérrez et al. [47] for birds and Barone [45] for mammals.

### Roadkill estimates and population effects

The death rate is higher than observed by simply counting corpses from a moving vehicle as the detectability of a carcass depends on fieldworker ability, animal size, survey method, weather conditions and traffic volume [48–51]. Furthermore, some animals undoubtedly moved off the road after being injured, were disintegrated after being repeatedly run over, or were removed by scavengers, and consequently were not recorded on surveys [48,49,52]. As the magnitude of this effect is unknown, we estimated the total number of roadkills for each taxon, depending on their persistence on the road. We used the formula provided by Gerow et al. [53] to obtain a more accurate, but still rough, mortality estimation.

M=(C/nS)*(I/Tp)

Where M is the estimated mortality; C is the number of carcasses found; *n*S is the number of surveys conducted; I is the number of days between surveys; T is the persistence time of each taxa; and *p* is the probability of detection. We employed two mean probabilities of detection, 67% for large animals (>100 g) and 27% for small animals (<100 g) based on data obtained in the literature [49–51,53–55], and specific carcass persistence times obtained by Santos et al. [52]. We calculated the minimum number of roadkills/km was calculated by dividing the observed roadkill count data by the total length of surveyed roads. We calculated a specific annual mortality estimate for each species and season, and an overall insular estimate (by summing all specific estimates). These estimates of road mortality are therefore conservative and should be considered with caution.

To roughly evaluate the roadkill effect on island populations of the most affected breeding bird species (>5 victims) and the threatened Houbara Bustard, we calculated the percentage of the affected population considering both the number of detected carcasses and the estimations corrected using the detection probabilities and persistence times (see above).

### Data analysis

Generalized linear models (GLM) with binomial error distribution and logit link function (logistic regressions) were used to study roadkill occurrence. As a response variable, we used a binomial variable (0 = randomly selected points or pseudo-absences; and 1 = roadkill locations). As only presence data (roadkill locations) was available in our original data set, we randomly selected 700 points (pseudo-absences) on the studied roads to compare the characteristics of roads where roadkills were recorded. Four explanatory variables were employed to describe the locations (i.e. randomly selected locations and real casualty location): 1) speed limit; 2) traffic volume; 3) distance to house; and 4) land uses (a three-level factor of vegetation and urban areas) in the surroundings. Speed limits were registered according to road signals during the fieldwork. Traffic volume refers to cars per day. Traffic volume data were from 2010 and are available at the local government website [43]. We obtained land uses and vegetation types from vegetation maps [41], and categorized them into three main groups to perform the analysis: natural (sandy, grassland, badlands, spurge bushes, etc.), exotic shrubland (mainly *Opuntia* cactus) and urban areas. Road width is also an important factor influencing the occurrence of road casualties [6,56]. However, Lanzarote roads have a low variation in width (8–15 m), so this variable was not considered in the analysis.

To study abundance of casualties, we divided the surveyed roads into sections according to the same speed limit and made buffer polygons of 50 m on each side of the roads. We used the total number of roadkills (i.e. including mammals and birds), and the number of mammalian and avian roadkills on the road sections as response variables. Five explanatory variables were calculated to characterize each polygon (road section): 1) the length of the road; 2) speed limit; 3) traffic volume; 4) the percentages of surface area covered by urban areas; and 5) by the secondary scrubland vegetation [41]. To analyse the factors affecting the number of roadkills (total, mammalian or avian roadkills) in each road section (response variable), we used GLMs with negative binomial error distributions and log link functions, and with the previous five explanatory variables.

Geographical analyses were conducted in QGIS v2.16.3. Models were fitted in R (version 3.3.32) using the ‘glm’ and ‘glm.nb’ functions of the stats and MASS packages, respectively. Explanatory variables were centred and scaled prior modeling with the ‘scale’ function. Models were compared to null models, i.e. including only the intercept, using the ‘anova’ function (stats package) and assumptions were checked using diagnostic plots (see S1 Text). Given the descriptive aim of our study, we did not conduct any model selection.

## Results

### Species affected

We found a total of 666 carcasses during the entire annual cycle (2.76 victims/km) belonging to a minimum of 37 taxa of mammals and birds (S1 Table). Mammals were the most abundant roadkills (412 [61.9% of those recorded]) and were represented by six introduced non-native species (five families) (S1 Table). The most common mammals were the Algerian Hedgehog *Atelerix algirus* (*n* = 156), the Feral Cat *Felis catus* (*n* = 124) and the European Rabbit *Oryctolagus cuniculus* (*n* = 105) comprising 93.4% of the total mammals.

Birds were represented by a minimum of 19 families and 254 individuals (S1 Table). The most affected families were: Columbidae (*n* = 51; 20.1% of birds), Burhinidae (*n* = 31; 12.6%), Ardeidae (*n* = 21; 8.3%), Laniidae (*n* = 15; 5.9%) and Passeridae (*n* = 11; 4.3%). Ruling out some rock pigeons *Columba livia*, in which feral and domestic individuals are difficult to distinguish, no captive or domestic bird species were recorded as roadkills. Except for Common Ringed Plover *Charadrius hiaticula*, Yellow Wagtail *Motacilla flava*, European Robin *Erithacus rubecula*, Willow Warbler *Phylloscopus trochilus* and European Pied Flycatcher *Ficedula hypoleuca*, all of the birds belonged to breeding species. Some bird species of conservation concern or with a relatively low population size were affected, such as Cattle Egret *Bubulcus ibis* (*n* = 21), Eastern Canary Islands Kestrel *Falco tinnunculus dacotiae* (*n* = 7), Houbara Bustard *Chlamydotis undulata fuertaventurae* (*n* = 2), Eurasian Stone Curlew *Burhinus oedicnemus insularum* (*n* = 32), Barn Owl *Tyto alba gracilirostris* (*n* = 6) and Southern Shrike *Lanius meridionalis* (*n* = 15) (S1 Table).

### Roadkill estimates and percentage of local population affected

Using the detection probabilities and specific carcass persistence times, the estimated number of vertebrates dead on the road each year was 9,664 (39.84 victims/km), with a total of 7,124 bird victims (29.49 individuals/km) and 2,540 mammals (10.51 individuals/km) (S1 Table). For three species (*Tyto alba*, *Bubulcus ibis*, and *Lanius meridionalis*), mortality estimations exceeded 25% of their insular breeding population sizes (Table 1).

**Table 1:** Percentage of population annually affected by roadkills on Lanzarote, Canary Islands, of some representative species. Species were selected according to number of carcasses and mortality estimations. Population sizes (individuals) were taken from literature [57–59].

Species	Population size	% of population affected using	
		Carcasses	Mortality
*Tyto alba*	160–200	3,0–3,8	83,8–117,5
*Bubulcus ibis*	350–400	5,3–6,0	43,7–101,7
*Lanius meridionalis*	1175–3116	0,5–1,3	26,8–71,1
*Upupa epops*	587–2772	0,3–1,4	16,1–76,0
*Falco tinnunculus*	300–400	1,8–2,3	9,8–13,0
*Burhinus oedicnemus*	485–3972	0,8–6,6	4,5–36,9
*Larus michahellis*	3370–3870	0,3–0,3	1,6–1,8
*Chlamydotis undulata*	334–579	0,3–0,6	1,2–2,1

### Factors affecting road casualties

**Fig 2:**
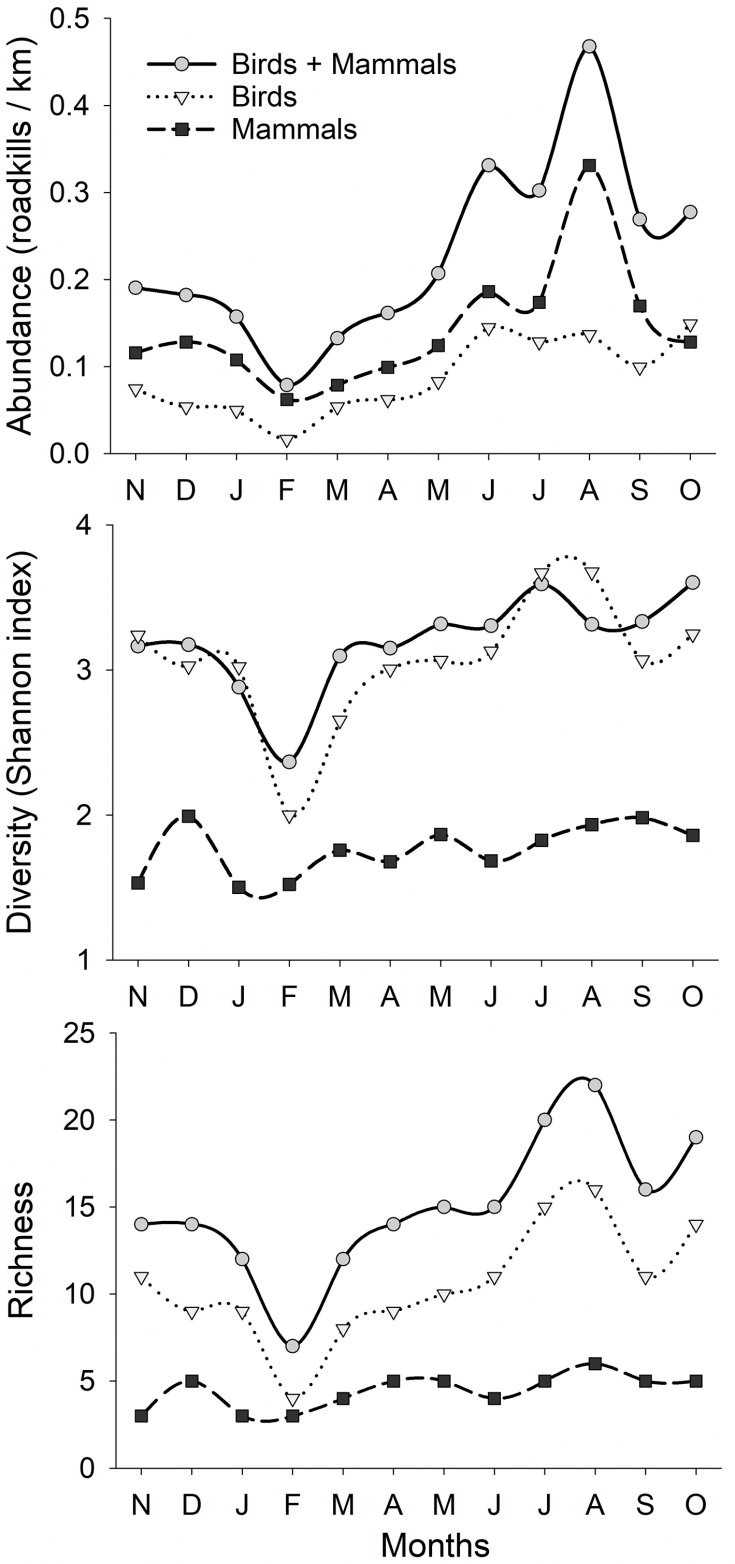
Seasonal variation of abundance (roadkills/km), diversity and richness of road casualties of wildlife on Lanzarote, Canary Islands, during November 2010 to October 2011.

Seasonal abundance of roadkills showed a high peak during the summer months for both mammals and birds (see Fig 2). Richness and diversity (Shannon index) of roadkill species varied among months, being higher in the summer for both mammals and birds (Fig 2). The roadkills were scattered along the studied roads, and only some sectors in the central part of the island seemed to have lower densities of casualties (Fig 1).

Binomial GLMs highlighted the most important variables explaining the probability of occurrence of a road casualty, and few differences were found between mammals and bird casualties (see Table 2). The model including all victims (mammals and birds) indicated that accidents occurred close to houses and in road sectors with higher speed limits. However, mammal kills occurred in road sectors close to houses, with high speed limits and in areas surrounded by exotic bushes, while birds appeared in road sectors close to houses, with high speed limits and low traffic volume (Table 2). The negative binomial GLMs built to evaluate factors affecting abundance of road casualties indicated that the speed limit and length of road section are the unique significant variables (Table 3).

**Table 2:** Results of the generalized linear models built to evaluate the occurrence of roadkills on Lanzarote, Canary Islands. Significant variables are in bold, i.e. 95% Confidence Intervals (CI) do not include zero.

Model	Estimate	Std. Error	lower 95% CI	upper 95% CI
***Total***				
(Intercept)	-0.173	0.142	-0.452	0.104
Exotic shrubland	0.251	0.165	-0.072	0.575
Urban	0.044	0.174	-0.298	0.386
**Speed limit**	**0.164**	**0.060**	**0.048**	**0.282**
Traffic volume	-0.112	0.059	-0.229	0.002
**Distance to house**	**-0.168**	**0.065**	**-0.300**	**-0.044**
***Mammals***				
**(Intercept)**	**-0.717**	**0.167**	**-1.049**	**-0.394**
**Exotic shrubland**	**0.392**	**0.192**	**0.019**	**0.771**
Urban	0.043	0.205	-0.356	0.447
**Speed limit**	**0.153**	**0.068**	**0.021**	**0.286**
Traffic volume	-0.051	0.064	-0.179	0.072
**Distance to house**	**-0.142**	**0.074**	**-0.295**	**-0.002**
***Birds***				
**(Intercept)**	**-1.057**	**0.187**	**-1.432**	**-0.697**
Exotic shrubland	0.018	0.221	-0.411	0.454
Urban	0.053	0.229	-0.392	0.506
**Speed limit**	**0.175**	**0.080**	**0.018**	**0.333**
**Traffic volume**	**-0.224**	**0.089**	**-0.407**	**-0.058**
**Distance to house**	**-0.201**	**0.094**	**-0.398**	**-0.029**

**Table 3:** Results of the generalized linear models built to evaluate the factors affecting the abundance of wildlife (mammals and birds) roadkills on Lanzarote, Canary Islands. Significant variables are in bold, i.e. 95% Confidence Intervals (CI) do not include zero.

Model	Estimate	Std. Error	lower 95% CI	upper 95% CI
***Total***				
**(Intercept)**	**0.902**	**0.078**	**0.750**	**1.055**
**Speed limit**	**0.261**	**0.088**	**0.078**	**0.445**
Traffic volumen	-0.015	0.081	-0.181	0.158
**Length**	**0.281**	**0.077**	**0.128**	**0.457**
% Secondary scrublands	0.078	0.111	-0.143	0.292
% Urban	-0.181	0.120	-0.419	0.049
***Mammals***				
**(Intercept)**	**0.477**	**0.088**	**0.305**	**0.650**
Speed limit	0.203	0.100	-0.006	0.414
Traffic volumen	0.006	0.091	-0.180	0.198
**Length**	**0.297**	**0.086**	**0.131**	**0.490**
% Secondary scrublands	0.239	0.131	-0.012	0.486
% Urban	0.009	0.142	-0.261	0.277
***Birds***				
(Intercept)	0.025	0.102	-0.176	0.225
**Speed limit**	**0.289**	**0.116**	**0.053**	**0.529**
Traffic volume	-0.134	0.112	-0.374	0.103
**Length**	**0.243**	**0.097**	**0.050**	**0.467**
% Secondary scrublands	-0.111	0.142	-0.399	0.170
% Urban	-0.196	0.151	-0.501	0.102

## Discussion

### General considerations

We quantified the impact of road mortality on vertebrates on Lanzarote. During an entire annual cycle, we recorded a total of 2.76 roadkills per km, but considering survey biases and carcass persistence, the actual figure could be close to 40 (S1 Table). For some threatened bird species (e.g., Houbara Bustard, Eurasian Stone-Curlew and Barn Owl), the impact of road casualties suggests that this mortality source may have demographic effects on the island population. The main road features explaining roadkills were different for native birds vs. introduced mammals. Mammal kills usually occurred in sectors with high speed limits, close to houses and surrounded by exotic bushes, while birds appeared mainly in road sectors with high speed limits, close to houses and with low traffic volume.

Our annual estimates of mortality should be considered as indicative since they may be biased (detectability and carcass persistence times were obtained from the literature) and only part of the island road network was surveyed (52.8%). The detectability of the smallest species is low when roadkill surveys are conducted by car; this is true of amphibians, lizards and passerines. In this regard, no individuals of Atlantic Lizard *Gallotia atlantica*, Canarian Shrew *Crocidura canariensis* or House Mouse *Mus domesticus* were found, although they are relatively common species on the island [24,60], and the number of passerines was also relatively low (S1 Table).

Removal of carcasses by scavengers could greatly influence studies on mortality rates and its causes, making them difficult to interpret. In Lanzarote, the Yellow-legged gull *Larus michahellis* (a common and widespread species) and the Common Raven *Corvus corax* [57] remove corpses as they are basically opportunistic feeders and scavenger species [61,62], however the rate of consumption is unknown. Despite these drawbacks, these results constitute an important piece of information for the Canaries, as it is the first detailed road mortality study for any of the Canary Islands ecosystems.

### Species affected

According to our annual mortality estimates, mammals are less affected than birds. However, as mentioned above, some biases may occur with small rodents and shrews [48,49]. The results indicated that the Algerian Hedgehog, the Feral Cat and the European Rabbit are highly affected by roadkill in Lanzarote, as occurs in other areas of Europe, perhaps related to their high local abundance and nocturnal behaviour [9,63]. There is strong evidence that these introduced species produce important negative ecological effects in the Canary Islands [64,65], thus, road mortality could be helping to minimize them. Finally, it is important to note that the high numbers of domestic dogs (*Canis lupus familiaris*) and feral cats are of socioeconomic and safety concern (a total of 133 individuals of both species); their sizes are sufficiently large to produce fatal traffic accidents [9].

Regarding the bird roadkills, passerines were probably underestimated because of their low detection probability and persistence time, and accordingly many of them remained unidentified as they were totally destroyed. As a whole, the most abundant group was made up of urban species (pigeons, dove, sparrows, etc.), followed by nocturnal species such as Stone Curlews and Barn Owls (S1 Table). In this sense, the low number (only two) of Procellariformes (petrels and shearwaters) found in this study is intriguing. Fledglings of burrow-nesting seabirds are attracted to and disoriented by artificial lights and then forced to land when they fly at night, thus being prone to vehicle collisions [31]. Each year, in October and November, coinciding with the Cory’s Shearwater (*Calonectris diomedea*) fledging period, local authorities conduct a rescue campaign to mitigate mortality of the young birds grounded by light pollution (~100 individuals) and release them to the sea.

### Seasonality of roadkills

We found slight monthly differences in mortality rates, but the highest number of roadkills was concentrated in the summer months (from June to September) (Fig 2). We should note that many species breed during the spring [46], and consequently, the summer is a period when population increases due to offspring and mortality increases due to inexperience of juveniles. Many studies have demonstrated that juvenile dispersion or migratory phenology greatly influence the number of road casualties [30,66–68]. In Lanzarote, some migratory species (*Charadrius hiaticula*, *Motacilla flava*, *Erithacus rubecula*, *Phylloscopus trochilus* and *Ficedula hypoleuca*) were recorded in low numbers (a total of 11 carcasses) during autumn (October-December; S1 Table), coinciding with the post-breeding passage time in the Canary Islands [46].

### Factors affecting roadkills

Binomial GLMs indicated differences in the road features affecting mammal and bird road casualties. For mammals, roads with higher speed limits and surrounded by transformed areas close to houses had a higher probability of roadkills. Mammal densities are probably higher in exotic shrubland areas, especially on a xeric island like Lanzarote, but no studies are available on local habitat selection of these species. The models suggest that bird roadkills occur in road sectors with high speed limits, low traffic volume and close to houses (Table 2). These results could be influenced by the large number of pigeons *Columba livia*, doves *Streptopelia* spp. and Spanish Sparrows *Passer hispaniolensis* (*n* = 65) recorded (~ 25% of total bird carcasses) that are tame species strongly associated with towns and urbanized areas [46].

Usually, in arid areas primary production on road verges is higher than in adjacent rangelands due to their particular management regimen. The local government of Lanzarote is developing an embellishment plan for road verges that consists of planting ornamental shrubs or palms, as they are usually the unique green/living vegetation within the surroundings. Thus, many species may be attracted to them for foraging due to the high density of food resources [69–73]. As a result, they are more prone to vehicle collisions [74]. The high numbers of Cattle Egret *Bubulcus ibis* and Eurasian Stone Curlew *Burhinus oedicnemus insularum* carcasses are related to their foraging behaviour, as both often utilize road verges (*pers*. *obs*.). Other species such as Common Kestrel, Barn Owl and Southern Shrike often hunt close to roads (Dean and Milton, 2003; Grilo et al., 2012), and thus, it has been suggested that vehicle collisions of these species are highly influenced by roadside perch availability [56]. Finally, scavengers are usually attracted to roads to consume recently dead animals [5,70]; this may explain the gulls (*Larus* spp.) recorded in our study.

### Impacts on populations and conservation remarks

Detecting the key factors involved in wildlife mortality due to wildlife-vehicle collision is a prerequisite for effective mitigation measures [75–80]. Roads, traffic, transmission towers, artificial lights and other infrastructure related to road network features have rapidly increased in the Canary Islands in the last four decades [34], and will probably continue increasing. As a result, roadkills will continue to affect wildlife, especially the most susceptible species. The annual estimates of mortality suggest that vehicle collisions play an important role in the population dynamics of some rare or threatened species (Table 1; S1 Table). Lanzarote is host to apparently healthy populations of Cattle Egret, Eurasian Stone Curlew and Southern Shrike [57], but the high road mortality rates suggest the existence of some negative demographic effects. Our results also indicate that road mortality is an important threat, unidentified until now, for the endangered Houbara Bustard [59], which is also severely affected by collisions with other infrastructures like telephone and power lines [27,28]. Moreover, estimates quantitatively confirm that the population of the threatened endemic subspecies Barn Owl is suffering a high impact [81]. These two taxa are of conservation concern as they are endemic subspecies with limited distribution and discrete population sizes [57]. Thus, the inclusion of this non-natural source of mortality in management strategies could be crucial for their conservation.

### Conclusions

Our findings highlight four key messages: (1) although raw data indicate that mammals were more affected than birds, mortality estimates that consider detectability and persistence time of carcasses indicate the opposite; (2) road mortality was higher during the summer, when inexperienced juveniles increase population numbers; (3) modeling demonstrated that traffic characteristics (speed limit and traffic volume) and habitat features (presence of transformed areas and proximity to houses) were major contributors to the deadliest road segments; and 4) some species of conservation concern are affected by road mortality (e.g., Houbara Bustard, Eurasian Stone-Curlew and Barn Owl), and thus precise studies are required to evaluate the possible demographic effects on populations. Given that the Canary Islands are considered one of the most important biodiversity hotspots within Europe [19], more studies on the impact of infrastructure overall and in particular the road network on the wildlife of other islands and habitats are needed to establish proper management strategies.

## Supporting information

S1 Table Number of carcasses (roadkills) found on Lanzarote, Canary Islands (November 2010-October 2011). Mortality was estimated by considering carcass removal time (PT) and probability of observer detectability (D) of each species according to bibliography [52].
(DOCX)

S1 Text Model assumptions and validations. (DOCX)

S1 File Species, locations (UTM 28R coordinates) and dates of roadkills found on Lanzarote, Canary Islands (November 2010-October 2011). (CSV)
