# Supplementary material for: Wildlife-vehicle collisions in Lanzarote Biosphere Reserve, Canary Islands
Source: PLoS One. 2018 Mar 21;13(3):e0192731. doi: 10.1371/journal.pone.0192731 (PMC5862401; doi:10.1371/journal.pone.0192731)
Supplement: S1 Table — Mortality was estimated by considering carcass removal time (PT) and probability of observer detectability (D) of each species according to bibliography [52]. (DOCX) [file pone.0192731.s001.docx]

**S1 Table**

Wildlife-vehicle collisions in Lanzarote Biosphere Reserve, Canary Islands

Gustavo Tejera^1^, Beneharo Rodríguez^1*^, Carlos Armas^2^& Airam Rodríguez^3^

^1^ *Canary Islands’ Ornithology and Natural History Group (GOHNIC), La Malecita s/n, 38480 Buenavista del Norte, Tenerife, Canary Islands, Spain*

^2^*Piedra Viva 26, 35559 San Bartolomé, Lanzarote, CanaryIslands, Spain*

^3^*Department of Evolutionary Ecology, Estación Biológica de Doñana (CSIC), Avda. Américo Vespucio 26, 41092 Seville, Spain*

*corresponding author: Beneharo Rodríguez, email: benerguez@gmail.com

**Number of carcasses (roadkills) found on Lanzarote, Canary Islands (November 2010-October 2011). Mortality was estimated by considering carcass removal time (PT) and probability of observer detectability (D) of each species according to bibliography [1]**

| Species | Roadkills | | | | | PT | D | Mortality |
| --- | --- | --- | --- | --- | --- | --- | --- | --- |
|  | Autumn | Winter | Spring | Summer | Total | (days) | (%) |  |
| Fam. Phasianidae |  |  |  |  |  |  |  |  |
| *Alectoris barbara* | 0 | 0 | 0 | 5 | 5 | 4 | 67 | 28 |
| Fam. Procellariidae |  |  |  |  |  |  |  |  |
| *Bulweria bulwerii* | 0 | 0 | 0 | 2 | 2 | 1 | 27 | 112 |
| Fam. Ardeidae |  |  |  |  |  |  |  |  |
| *Bubulcus ibis* | 5 | 2 | 8 | 6 | 21 | 4 | 67 | 118 |
| Fam. Falconidae |  |  |  |  |  |  |  |  |
| *Falco tinnunculus* | 2 | 3 | 0 | 2 | 7 | 4 | 67 | 39 |
| Fam. Otidae |  |  |  |  |  |  |  |  |
| *Chlamydotis undulata* | 0 | 0 | 2 | 0 | 2 | 6 | 67 | 7 |
| Fam. Burhinidae |  |  |  |  |  |  |  |  |
| *Burhinus oedicnemus* | 9 | 6 | 10 | 7 | 32 | 4 | 67 | 179 |
| Fam. Charadriidae |  |  |  |  |  |  |  |  |
| *Charadrius hiaticula* | 1 | 0 | 0 | 0 | 1 | 1 | 27 | 56 |
| *Charadrius* sp. | 1 | 0 | 0 | 0 | 1 | 1 | 27 | 56 |
| Fam. Laridae |  |  |  |  |  |  |  |  |
| *Larus fuscus* | 0 | 0 | 1 | 0 | 1 | 4 | 67 | 6 |
| *Larus michahellis* | 1 | 1 | 5 | 4 | 11 | 4 | 67 | 62 |
| Fam. Columbidae |  |  |  |  |  |  |  |  |
| *Columba livia* | 0 | 2 | 10 | 8 | 20 | 4 | 67 | 112 |
| *Streptopelia decaocto* | 0 | 0 | 5 | 7 | 12 | 1 | 67 | 269 |
| *Streptopelia turtur* | 0 | 1 | 1 | 0 | 2 | 1 | 67 | 45 |
| *Streptopelia* sp. | 4 | 1 | 5 | 7 | 17 | 1 | 67 | 381 |
| Fam. Tytonidae |  |  |  |  |  |  |  |  |
| *Tyto alba* | 3 | 0 | 0 | 3 | 6 | 1 | 67 | 134 |
| Fam. Upupidae |  |  |  |  |  |  |  |  |
| *Upupa epops* | 1 | 5 | 1 | 1 | 8 | 1 | 27 | 446 |
| Fam. Alaudidae |  |  |  |  |  |  |  |  |
| *Callandrella rufescens* | 0 | 0 | 0 | 3 | 3 | 1 | 27 | 167 |
| Fam. Motacillidae |  |  |  |  |  |  |  |  |
| *Anthus berthelotii* | 1 | 1 | 0 | 1 | 3 | 1 | 27 | 167 |
| *Motacilla flava* | 1 | 0 | 0 | 0 | 1 | 1 | 27 | 56 |
| Fam. Turdidae |  |  |  |  |  |  |  |  |
| *Erithacus rubecula* | 2 | 0 | 0 | 0 | 2 | 1 | 27 | 112 |
| Fam. Sylviidae |  |  |  |  |  |  |  |  |
| *Sylvia conspicillata* | 3 | 0 | 0 | 0 | 3 | 1 | 27 | 167 |
| *Sylvia* sp. | 0 | 0 | 0 | 1 | 1 | 1 | 27 | 56 |
| *Phylloscopus trochilus* | 3 | 0 | 0 | 0 | 3 | 1 | 27 | 167 |
| Fam. Muscicapidae |  |  |  |  |  |  |  |  |
| *Ficedula hypoleuca* | 4 | 0 | 0 | 0 | 4 | 1 | 27 | 223 |
| Fam. Laniidae |  |  |  |  |  |  |  |  |
| *Lanius meridionalis* | 5 | 1 | 2 | 7 | 15 | 1 | 27 | 836 |
| Fam. Passeridae |  |  |  |  |  |  |  |  |
| *Passer hispaniolensis* | 1 | 2 | 4 | 4 | 11 | 1 | 27 | 613 |
| Fam. Fringillidae |  |  |  |  |  |  |  |  |
| *Bucanetes githagineus* | 1 | 1 | 0 | 1 | 3 | 1 | 27 | 167 |
| Passerine | 11 | 0 | 7 | 14 | 32 | 1 | 27 | 1,784 |
| Unidentified | 8 | 3 | 9 | 5 | 25 | 1 | 67 | 560 |
| TOTAL birds |  |  |  |  | 254 |  |  | 7,124 |
| Fam. Erinaceidae |  |  |  |  |  |  |  |  |
| *Atelerix algirus* | 25 | 25 | 40 | 66 | 156 | 4,5 | 67 | 776 |
| Fam. Canidae |  |  |  |  |  |  |  |  |
| *Canis lupus familiaris* | 3 | 0 | 3 | 3 | 9 | 9 | 67 | 22 |
| Fam. Felidae |  |  |  |  |  |  |  |  |
| *Felis catus* | 31 | 22 | 33 | 38 | 124 | 9 | 67 | 308 |
| *Mustela furo* | 1 | 0 | 0 | 0 | 1 | 1 | 67 | 22 |
| Fam. Muridae |  |  |  |  |  |  |  |  |
| *Rattus* sp. | 0 | 1 | 0 | 3 | 4 | 1 | 67 | 90 |
| Fam. Leporidae |  |  |  |  |  |  |  |  |
| *Oryctolagus cuniculus* | 28 | 12 | 20 | 45 | 105 | 2 | 67 | 1,175 |
| Unidentified | 2 | 0 | 3 | 8 | 13 | 2 | 67 | 146 |
| TOTAL mammals |  |  |  |  | 412 |  |  | 2,540 |
| TOTAL |  |  |  |  | 666 |  |  | 9,664 |

References

1. Santos SM, Carvalho F, Mira A. How long do the dead survive on the road? Carcass persistence probability and implications for road-kill monitoring surveys. PLoS ONE. 2011;6. doi:10.1371/journal.pone.0025383
