# Supplementary material for: Wildlife-vehicle collisions in Lanzarote Biosphere Reserve, Canary Islands
Source: PLoS One. 2018 Mar 21;13(3):e0192731. doi: 10.1371/journal.pone.0192731 (PMC5862401; doi:10.1371/journal.pone.0192731)
Supplement: S1 Text — (DOCX) [file pone.0192731.s002.docx]

**S1 Text**

Wildlife-vehicle collisions in Lanzarote Biosphere Reserve, Canary Islands

Gustavo Tejera^1^, Beneharo Rodríguez^1*^, Carlos Armas^2^& Airam Rodríguez^3^

^1^ *Canary Islands’ Ornithology and Natural History Group (GOHNIC), La Malecita s/n, 38480 Buenavista del Norte, Tenerife, Canary Islands, Spain*

^2^*Piedra Viva 26, 35559 San Bartolomé, Lanzarote, Canary Islands, Spain*

^3^*Department of Evolutionary Ecology, Estación Biológica de Doñana (CSIC), Avda. Américo Vespucio 26, 41092 Seville, Spain*

*corresponding author: Beneharo Rodríguez, email: benerguez@gmail.com

**Model assumptions and validations**

For occurrence models (generalized linear models with binomial distributions of errors and logit link functions), we checked QQ-plots and residuals versus predicted values through a simulation-based approach using the ‘DHARMa’ package (Hartig, 2016). We produced scaled residuals by simulating from the fitted model 9,999 runs and we created qq-uniform plots to detect deviations from overall uniformity of the residuals and plots of residuals against predicted values (Figures S1-S3). In addition, we compared full models to null models (i.e. only including the intercept) using the ‘anova’ function (stats package). Despite the small proportion of deviance explained (1.0 % for allroad kills, 1.2 % for Mammals, and 1.2 % for Birds), all models were significant when compared to null models (all road kills:*χ*^2^= 18.401, *df* = 5, *P* = 0.002; mammals: *χ*^2^= 17.311, *df* = 5, *P* = 0.004; birds: *χ*^2^= 12.772, *df* = 5, *P* = 0.026).Variance inflation factors (VIF) were calculated with the ‘VIF’ function of the ‘DescTools’ package, and they were not an issue (all VIF values < 1.27).

For abundance models, we started modeling by using a Poisson distribution of errors with log link function, but we found overdispersion in our data (dispersion parameters> 3). For that reason, we employed generalized linear models with negative binomial distribution of errors and log link functions for modeling, and we got smaller dispersion parameters (1.19, 1.28 and 1.05 for all road kills, mammals and birds, respectively). Overdispersion was calculated following (Zuur, Hilbe, & Ieno, 2013).We checked the assumptions of the negative binomial GLMs by inspecting the scatterplots of residuals plotted against fitted values (Figures S4-6), and explanatory variables. No obvious patterns were detected. All variance inflation factors (VIF) were < 2.66 and full models were highly significant in comparison to null models (all road kills:*χ*^2^= 45.952, *df* = 5, *P*< 0.001; mammals: *χ*^2^ = 44.794, *df* = 5, *P*< 0.001; birds: *χ*^2^ = 29.104, *df* = 5, *P*< 0.001).

**References**

Hartig, F. (2016). DHARMa: Residual Diagnostics for Hierarchical (Multi-Level/Mixed) Regression Models. Retrieved November 19, 2016, from https://cran.r-project.org/web/packages/DHARMa/index.html

Zuur, A. F., Hilbe, J. M., & Ieno, E. N. (2013). *A beginner’s guide to GLM and GLMM with R*. Newburgh, UK: Highland Statistics Ltd.


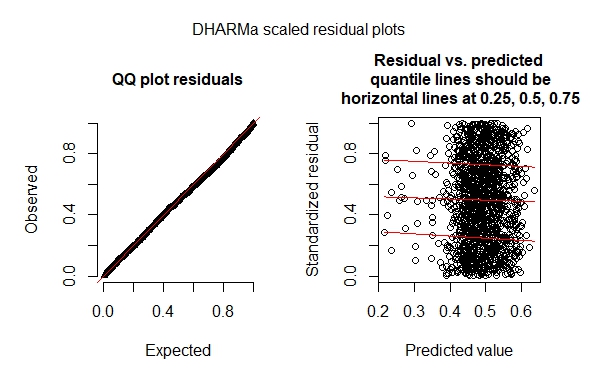


**Figure S1.** Diagnostic plots of the generalised linear model explaining the occurrence of all road kills (including birds and mammals) on Lanzarote, Canary Islands in relation to the four explanatory variables (speed limit; traffic volume; distance to house; and land uses (a three-level factor: urban, exotic shrubland, or natural vegetation).

**
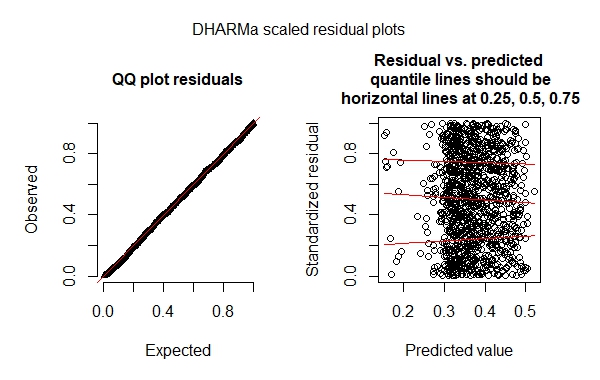
**

**Figure S2.** Diagnostic plots of the generalised linear model explaining the occurrence of mammalian road kills (including only mammal casualties) on Lanzarote, Canary Islands in relation to the four explanatory variables (speed limit; traffic volume; distance to house; and land uses (a three-level factor: urban, exotic shrubland, or natural vegetation).


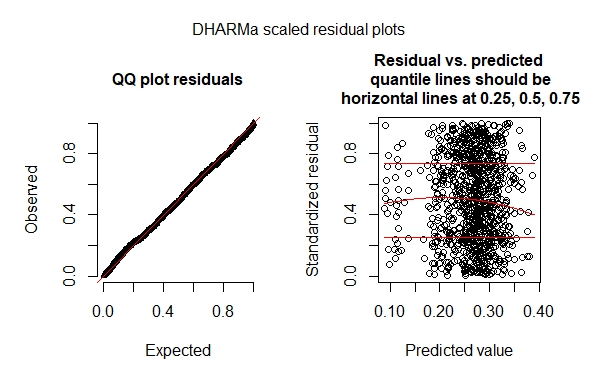


**Figure S3.** Diagnostic plots of the generalised linear model explaining the occurrence of avian road kills (including only bird casualties) on Lanzarote, Canary Islands in relation to the four explanatory variables (speed limit; traffic volume; distance to house; and land uses (a three-level factor: urban, exotic shrubland, or natural vegetation).

**
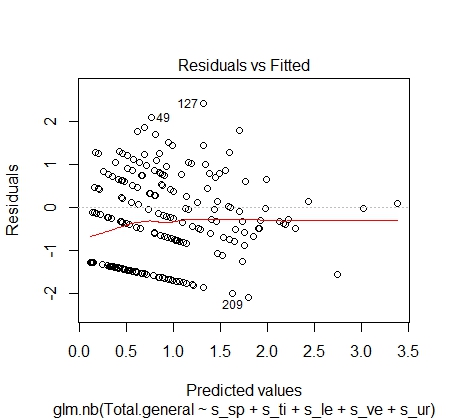
**

**Figure S4.** Scatterplots of residuals against fitted values for the generalised linear model explaining the abundance of all road kills (including mammal and bird casualties) on Lanzarote, Canary Islands in relation to the five explanatory variables (speed limit; traffic intensity; length of the road section; percentage of secondary scrubland vegetation; and percentage of urban area).

**
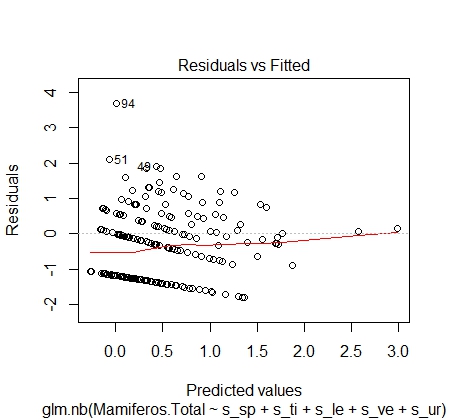
**

**Figure S5.**Scatterplots of residuals against fitted values for the generalised linear model explaining the abundance of mammalian road kills (including only mammal casualties) on Lanzarote, Canary Islands in relation to the five explanatory variables (speed limit; traffic intensity; length of the road section; percentage of secondary scrubland vegetation; and percentage of urban area).

**
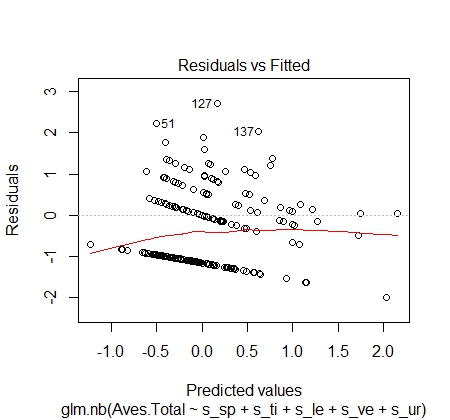
**

**Figure S6.**Scatterplots of residuals against fitted values for the generalised linear model explaining the abundance of avian road kills (including only bird casualties) on Lanzarote, Canary Islands in relation to the five explanatory variables (speed limit; traffic intensity; length of the road section; percentage of secondary scrubland vegetation; and percentage of urban area).
